# Ethnobotanical Study of Medicinal Herbs Used by the Luguru Tribe Against Various Human Ailments in Morogoro Rural District, Tanzania

**DOI:** 10.1155/tswj/6134739

**Published:** 2025-04-16

**Authors:** David Sylvester Kacholi

**Affiliations:** Department of Biological Sciences, Dar es Salaam University College of Education, University of Dar es Salaam, Dar es Salaam, Tanzania

**Keywords:** ethnobotany, ethnomedicine, fidelity level, herbal remedies, medicinal plants, use values

## Abstract

Since time immemorial, Tanzanians, particularly the Luguru tribe, have utilised medicinal herbs (MHs) to manage various ailments. However, few ethnobotanical studies have been conducted to document and quantitatively analyze them. This study documents and quantitatively analyzes MHs used by the Luguru people of Morogoro Rural District to address various ailments. The research was conducted between September 2022 and October 2023. Ethnobotanical data on MH were collected from 100 informants identified through the snowball method, employing semistructured interviews, focus group discussions, and field walks. A one-way analysis of variance (ANOVA) and an independent samples *t*-test were employed to examine statistically significant differences among social demographic variables. Quantitative indices, including family use value (FUV), MH use value (UV), fidelity level (FL), plant part value (PPV), and informant agreement ratio (IAR), were computed. A total of 30 MHs belonging to 13 families were reported to be used for managing various ailments. Asteraceae was the most represented family (seven species, FUV = 2.23). *Vernonia amygdalina* Del. (Asteraceae) had the highest UV index (0.571), while the root was the most utilised plant part (0.692). Malaria and fever (0.96) and metabolic disorders (0.94) had the highest IAR. The preferred modes of preparation and administration were decoction and oral, respectively. Female, illiterate, and elderly informants possessed significantly higher medicinal knowledge. The study demonstrates that the Luguru people possess a rich understanding of MHs and continue to rely on them to treat various ailments. The MHs with high UV, IAR, and FL can be a foundation for future phytochemical and pharmacological studies.

## 1. Introduction

Medicinal plant resources are rich in bioactive compounds that can be utilized to develop contemporary drugs for treating various ailments [1]. Since ancient times, medicinal herbs (MHs) have been traditionally used by different ethnic groups as a source of medication to manage multiple human health issues. They have served as an alternative remedy for many years, with countless contributions from traditional health practitioners to human health, especially as primary healthcare providers at the community level [2, 3]. Across the globe, traces of their use are evident, and even today, despite advancements in pharmacology, the use of MHs remains widespread in certain countries, particularly in middle- and low-income nations [4, 5]. In African countries, nearly 80% of the rural population relies on herbal remedies for primary healthcare [3, 6–8]. This reliance is primarily due to a strong belief in traditional remedies [9] and their relative accessibility, cost-effectiveness, and local availability compared to contemporary medicine [5, 10].

In Tanzania, about 80% of the population resides in rural areas, where forest resources are crucial for their livelihoods and where individuals primarily rely on traditional medicine for their healthcare needs [11, 12]. This dependence stems mainly from a strong cultural attachment to traditions, faith, the availability of MHs, and access to affordable traditional healers [12]. Additionally, the shortcomings of health facilities, the lack of healthcare providers, and the scarcity of modern medications, among the few available options, contribute to this reliance [13]. Traditional medicine in the country primarily centers on herbal remedies, often utilizing multiple species for various ailments. Most medicinal plants are sourced from forests, with few cultivated in farms or gardens [14, 15]. While many forests in Tanzania are protected, some unprotected areas still exist. Public forest lands are accessible for various uses, with resource harvesting regulated through licensing. However, many unprotected forests consist of Miombo woodlands, which supply food, fuel, construction materials, and medicine but face grave threats from overexploitation [16, 17]. In the Morogoro Rural District, where this study was conducted, deforestation has occurred due to the demand for agricultural land and charcoal production, leaving remaining forests unprotected and putting forest resources at risk [18].

MHs are essential to healthcare in the Morogoro Rural District and other settings [19, 20]. However, regrettably, like in other countries such as India [21, 22], Kenya [23], and Namibia [24], information on their traditional medicine of the Luguru has been dominated by oral tradition rather than documentation of the data for sustainability [25, 26]. Moreover, given the present deforestation rate, biodiversity loss, increasing urbanization, and disinterest of the young generation, accurate documentation of medicinal plants and associated traditional healing practices is paramount, as it is essential to try to circumvent the loss of knowledge by preserving information on useful MHs. Therefore, this study aimed to document and quantitatively analyze MHs used by the Luguru people living around the Kimboza Forest Reserve in Morogoro Rural District to manage various human ailments. Moreover, it is imperative to transform indigenous knowledge into a scientific perspective to enhance, conserve, and utilize it reasonably.

## 2. Materials and Methods

### 2.1. Description of the Study Area

**Figure 1:**
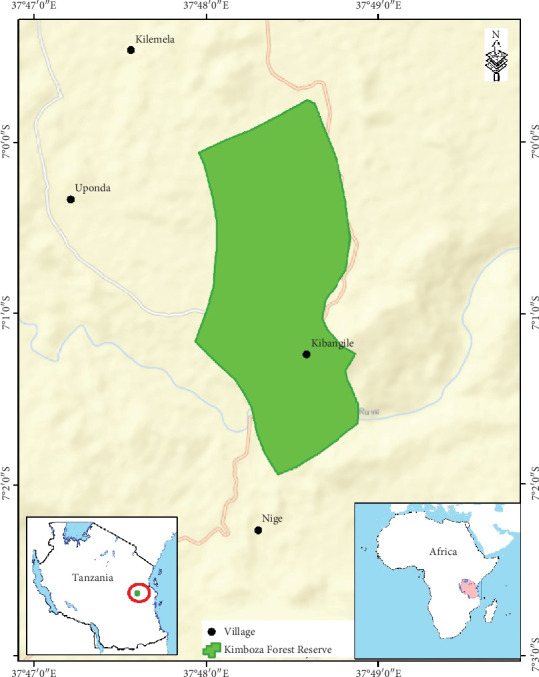
Map showing the study villages around Kimboza forest reserve.

The present study was conducted in the Morogoro Rural District in the Morogoro Region, Tanzania. The district is located about 260 km from Dar es Salaam City and covers an area of 19,056 km^2^. Its population is 387,736, of which 50.4 are females, and the average household size is 3.8 [27]. The Pwani Region borders the district to the north and east, the Kilombero District to the south, the Kilosa District to the southwest, and the Mvomero and Morogoro urban districts to the west. The district is inhabited by the Luguru ethnic group, also known as Waluguru in Swahili. The majority of the locals speak Luguru and Swahili languages. The present survey of MHs was conducted in four villages, namely, Kilemela, Uponda, Nige, and Kibangile, surrounding the Kimboza Forest Reserve in the district (Figure 1). The population of the four villages is estimated to be 6892 [24]. The forest reserve is situated 60 km from Morogoro Municipality between latitude 06°59⁣′ and 07°02⁣′ South and longitude 37°47⁣′ and 37° 49⁣′ East. It lies on the foothills of the Uluguru Mountains district, occupying an area of 405 ha with an altitudinal range of 300–400 m above sea level. The forest reserve is of particular conservation interest due to its exceptional biodiversity, including many endemic plants, animals, and 226 invertebrates [28, 29].

The study area's climate is oceanic due to its immediacy to the Indian Ocean, with a bimodal rainfall regime. The short rains last from October to December, while long rains last from March to May. The area's mean annual rainfall and temperature are 740 mm and 25.1°C, respectively. The locals in the villages around the forest engage in agricultural activities as their main socioeconomic activity. The locals living adjacent to the forest reserve have admittance rights over the forest reserve as specified in the forest management plan by-laws, and the locals living around it depend on it for collecting plant resources for medicinal purposes and other uses [18, 26].

### 2.2. Informant Sample Size Determination

The sample size of informants for gathering data on herbal medicine was determined according to Cochran's formula [30], as indicated below:

(1) $$\begin{array}{c} n=\frac{N}{1+N\;\left(e^{2}\right)}, \end{array}$$

whereby *n* is the informant sample size, *N* is the total number of households around the forest reserve, and *e* is the maximum margin of error (i.e., 5%). The total number of households was 133. Hence, the required informant size was 100:

(2) $$\begin{array}{c} n=\frac{133}{1+133\;\left(0.05^{2}\right)}=99.8\approx 100. \end{array}$$

Thus, among the selected informants were traditional healers (76%) and elders (24%), who are the custodians of information related to the application of MHs.

### 2.3. Ethnobotanical Data Collection

Ethnobotanical data were collected from September 2022 to October 2023 in the four villages surrounding the forest reserve. All herbal plants reported to be used as remedies were gathered during the survey. A combination of semistructured interviews, guided field walks, and market surveys was employed according to the specifications of the questionnaire. The snowball technique facilitated the identification of informants. Interviews were conducted with informants using a semistructured questionnaire in Swahili. The interview centered on herbal medicine, parts used, ailments treated, sources or habitats, and preferences for medicinal plants regarding their use, preparation, and application of remedies. Informants were encouraged to share their knowledge of herbal plants and their uses freely and without restriction. The respondent validation or member checking method was used to validate the informant's responses. Several MHs that were not identified in the field were collected for further identification and confirmation at the Herbarium of the Department of Biological Sciences at Dar es Salaam University College of Education (DUCE), where the specimens were ultimately stored. Only MHs confirmed by at least three informants were included in this study. All scientific names were confirmed through the Plants of the World Online database (https://powo.science.kew.org/). The survey data were presented in tables, graphs, and percentages.

### 2.4. Ethical Procedure

Before data collection, the informants were requested to provide voluntary oral informed consent. Each informant was also informed of the research goals and that the data obtained were for academic purposes. All informants provided verbal informed consent to participate in this study.

### 2.5. Data Analysis

Descriptive and quantitative statistical methods were used to analyze the respondents' sociodemographic information. A one-way analysis of variance (ANOVA) and an independent samples *t*-test were used to probe for significant differences within variables. Quantitative ethnobotanical tools such as relative frequency citation (RFC), family use value (FUV), use value (UV), plant part value (PPV), fidelity level (FL), and informant agreement ratio (IAR) were used for data analysis. All analyses were carried out using Microsoft Excel 2010 and QED statistics software.

#### 2.5.1. FUV

The FUV index was used to identify the significance of MP families. It is an index of cultural importance used in ethnobotany to calculate the value of biological plant taxon [31]. The index was calculated as per the following formula:

(3) $$\begin{array}{c} \text{FUV}=\frac{{\text{UV}}_{s}}{N_{s}}, \end{array}$$

where UV_*s*_ is the number of informants reporting the family and *N*_*s*_ is the total number of MPs within each family.

#### 2.5.2. UV

The UV index demonstrates the relative importance of MP known locally, reflecting the importance of each MP to informants [32]. It was calculated as per the following formula:

(4) $$\begin{array}{c} \text{UV}=\frac{U_{i}}{N}, \end{array}$$

where *U*_*i*_ is the number of use reports cited by each informant (*i*) for a given MP and *N* is the total number of informants interviewed for a given MP.

#### 2.5.3. PPV

The PPV index is normally computed to confirm the most utilized MP part [33]. The index was calculated using the following formula:

(5) $$\begin{array}{c} \text{PPV}=\frac{{\text{RU}}_{\text{plant⁣part}}}{\text{RU}}, \end{array}$$

where RU_plant⁣part_ is the sum of uses mentioned per part of the plant and RU is the number of uses of all plant parts. The MP part with the highest index value is the most utilized by the locals.

#### 2.5.4. FL

The FL computes the significance of a species for a given purpose. Most commonly used MPs have high FL values [34]. The FL values were computed using the following formula:

(6) $$\begin{array}{c} \text{FL}=\frac{I_{p}}{I_{u}}\times 100, \end{array}$$

where *I*_*p*_ is the number of informants who independently cited the importance of MP to cure a particular disease and *I*_*u*_ is the total number of informants who reported the plant to treat any given ailment.

#### 2.5.5. IAR

The IAR is usually used to identify the potentially effective MPs in a certain ailment category, or one can say the index is used to check informant agreement in the utilization of MPs in various ailment categories among informants in the study area [35]. The values for IAR range from 0 to 1. The IAR was computed as shown below:

(7) $$\begin{array}{c} \text{IAR}=\frac{N_{\text{ur}}-N_{t}}{N_{\text{ur}}-1}, \end{array}$$

whereby *N*_ur_ is the number of use reports in each ailment category and *N*_*t*_ is the total number of taxa used in each ailment category.

## 3. Results and Discussion

### 3.1. Sociodemographic Profile of Informants

Out of 100 interviewed informants, 58% were females, and 42% were males (Table 1). The females demonstrated a more excellent knowledge of herbal medicine than the male informants (*p* ≤ 0.001). This could be related to women being closer to the family members' welfare than men. A similar finding was reported in Bengkulu, Indonesia [36], the Western province of Austria [37], and Bahia, Brazil [38], where women had more medicinal plant knowledge than men because they were responsible for food and healthcare in their households. However, some ethnobotanical studies have indicated that men possess more excellent ethnomedicinal knowledge than women, as men are traditionally viewed as traditional healing practitioners. For instance, in northwestern Ethiopia, males are known for passing the traditional healing knowledge to the first son in a household; hence, they possess more medicinal plant knowledge than women [39]. A similar finding was also reported in northeastern Brazil [40].

In terms of age groups, the majority of the informants were of the age group of > 60 years (36%), followed by informants of age group 41–60 years (29%) and 20–40 years (Table 1). The elders (> 60 years) had significantly higher knowledge of herbal medicine than the young ones (*p* ≤ 0.001). This is because they hold more of the ancestral knowledge they have accrued through experience and interaction with the natural environment than the younger informants (Table 1). The relationship between age and knowledge of herbal medicine among the informants was strong and positively correlated (*r* = 0.994), indicating that knowledge of herbal medicine increases with age. A similar finding was also reported in other ethnobotanical studies in the Mara Region, Tanzania [15], India [21], Pakistan [41], and Ondo State, Nigeria [42]. The younger generation's lower knowledge of herbal medicine could be allied with various factors, such as disbelief, civilisation, and lack of interest [43, 44]. Similarly, the deterioration of traditional knowledge among younger generations has been reported in Indonesia [36], Mozambique [45], and Ecuador [46], where the young generation is more interested in modern medicine than traditional medicine, which they consider to be superstitious and primarily used by uneducated and poor people [47]. The finding indicates that traditional knowledge is at risk because oral transmission from elders to the younger generation can no longer be assured. Moreover, during the survey, some informants mentioned that they used to be familiar with numerous MHs for various ailments, but nowadays, they have forgotten some. This indicates a loss of traditional knowledge regarding the use of herbal medicine. Therefore, documenting and archiving valuable MHs are crucial.

Regarding education level, 44% of the informants were illiterate and had more knowledge of herbal medicine than the rest of the categories (Table 1). The informants with tertiary education levels had limited knowledge of herbal medicine. The difference between the levels and knowledge of herbal medicine was significant (*p* ≤ 0.001); the knowledge and use of herbal medicine declined with increasing education level (*r* = −0.993). The present results agree with the ethnobotanical studies conducted in Morocco Rif [33] and Nigeria [42]. A survey conducted in northeastern Brazil [48] reported that since less educated people tend to be out of the job market, they usually devote much of their time interacting with natural resources and, subsequently, more excellent knowledge about them.

### 3.2. Medicinal Plant Knowledge Acquisition

Most informants (58%) reported having acquired medicinal plant knowledge from family members, while others learnt the knowledge from herb doctors (20%), friends (12%), ancestor spirits (8%), and self-trained through readings (2%). A similar finding was reported in the Samburu community in Kenya [49] and Nyamwezi community in Tanzania [50], whereby family members and herbalists contributed significantly to the transfer of medicinal knowledge.

### 3.3. Habitats of Medicinal Herbs

In the present study, 47% of the informants reported collecting MHs from the wild (forest reserve and general lands), 30% from cultivated areas (home gardens and farmlands) and 23% from both wild and cultivated environments. The finding shows that the wild environment, especially the forest reserve, is essential for the livelihood of the locals in the study area. However, herbal medicine cultivation seems weak, and more exploitation pressure is on wild resources. This finding agrees with reports from the Suro Barguda District in Ethiopia [51] and the Tabora Region in Tanzania [12, 50], which explained that most medicinal plants are well adapted and available in wild environments but are highly affected due to overharvesting for diverse uses. Therefore, cultivating valuable MHs in farms and home gardens is highly recommended for sustainability.

### 3.4. Most Used Families and FUV

A total of 30 MHs belonging to 13 families were recorded for treating various human ailments in the study area. The family with the highest number of species was Asteraceae (seven species, 23.3% of the recorded medicinal species), followed by Solanaceae (five species, 16.6%), Fabaceae (four species, 13.3%), and Lamiaceae (three species, 10.0%). The remaining families were represented by two or one species (Table 2). Based on the FUV index (Table 2), the five families with the highest values are Asteraceae (2.23), Lamiaceae (1.53), Fabaceae (1.17), Solanaceae (1.10), and Euphorbiaceae (0.93). The possession of high value could be explained by the wide distribution of the families in the study area because of environmental factors favoring their species' development and adaptation. Similarly, a study conducted by [25] reported Fabaceae, Asteraceae, and Euphorbiaceae being the most speciose families in the same area.

### 3.5. UVs

To assess the relative importance of the recorded medicinal species, the UV index was computed, and values ranged from 0.060 to 0.570 (Table 2). The findings revealed that *V. amygdalina* had the highest UV index of 0.571, followed by *B. pilosa* (0.430) and *Ocimum suave* Willd. (Lamiaceae) (0.370), *Abrus precatorius* L. (Fabaceae) (0.320), and *Acalypha fruticosa* Forssk. (Euphorbiaceae) (0.320). The highest UV index was because a large number of informants cited these MHs. *B. pilosa* has been reported to possess various pharmacological activities, such as antibacterial [52], antifungal, hypotensive, vasodilatory [53], and wound healing [54, 55]. The bitter leaf juice or decoction of *V. amygdalina* has been reported to have several human health benefits for lowering high blood pressure, aiding weight loss, and enhancing women's fertility [56]. Therefore, MHs with high UV indexes must be further examined for phytochemical and pharmacological reasons to recognize their bioactive ingredients that can be used for drug formulation [57], and they should be prioritized for conservation as their preferred uses may endanger their existence due to overexploitation.

### 3.6. FL

The fidelity value (FL) is a *suitable index for identifying the most preferred medicinal plant used by the locals of a specific area to treat particular diseases effectively*. The FL in this study ranged from 81% to 100% (Table 3). The findings showed that six MH species, namely, *Justicia heterocarpa* L. (Acanthaceae), *Catharanthus roseus* (L.) G. Don (Apocynaceae), *Cucurbita moschata* Duchesne (Capparaceae), *A. fruticosa* (Euphorbiaceae), and *Smilax anceps* Wild. (Smilacaceae), had an FL of 100%. The FL of 100% designates that all use reports cited the same method for using the plant for management [58, 59]. The findings inform that the locals living in the study area depend on these MHs to manage the specified ailments. The highest FL determines the significance of an MH and the need for further investigation using biological, phytochemical, and pharmacological activities. Therefore, further investigation is required to evaluate the efficacy and authenticity of these MHs. Additionally, MH with low FL should be addressed but conserved to avoid increasing the risk of knowledge disappearing.

### 3.7. Ailment Categories and IAR

The IAR index relies on the availability of MHs to treat different ailments in a study area. The index ranges from 0 to 1. The high degree of agreement among informants indicates the effectiveness of various plant taxa in each ailment category, while the low values designate disagreement. The IAR has been vital for scrutinizing ethnobotanical data [60, 61]. In this study, the IAR values ranged from 0.82 to 0.96 (Table 4). The three ailment categories with the highest degree of agreement were Malaria and fever disorders (0.96), followed by metabolic disorders (0.94), gastrointestinal disorders (0.93), and dermatological and neurological disorders (with 0.90 each). The findings verified that the ailment recurrent in the study area has a higher IAR value. The higher IAR values show the rational trustworthiness of informants on the utilization of herbal remedies [62]. Also, the IAR values indicate that the locals share the therapeutic knowledge of the most important plant species for commonly occurring ailments in the study area. Hence, MHs with high IAR should be conserved and prioritized for phytochemical and pharmacological studies.

### 3.8. Plant Parts Used for Herbal Remedies

The locals in the study area harvest different medicinal plant parts to prepare herbal remedies. These parts include root, fruit, leaf, and bark. Based on the PPV index, the root was the dominant part used for treating human ailments in the study area (with PPV of 0.692), followed by leaf (0.538), bark (0.282), and fruit (0.128). Similarly, ethnobotanical studies conducted in Uganda [63], Ethiopia [64], and Zambia [24] indicated that roots are dominant in the preparation of herbal medicine. The preferential use of roots is due to the belief that the therapeutic power of MHs is limited to roots rather than other parts [3, 65]. Although roots are believed to comprehend extra potent pharmacological ingredients, their exploitation can destroy MHs and endanger their survival. Thus, using leaves as an alternative is advocated whenever possible because they are readily available, easily exploited and simple to formulate remedies [33]. Moreover, to ensure a sustainable supply of the MHs' resources, the study proposes the establishment of germplasm banks and conservation areas (in situ or ex situ) in the district and other parts of the country.

### 3.9. Methods of Preparing and Administering Remedies

Several preparation techniques, including decoction, infusion, crushing/pounding, concoction, and chewing, are used to prepare remedies (Table 2). The findings disclosed that most remedies were prepared from decoction (51.6%), followed by crushing/pounding (22.6%), infusion (16.1%), concoction (6.5), and chewing (3.2%). The decoction method is preferred because it helps to speed up the extraction of bioactive ingredients from the plant's materials, sterilizes used plant materials, and detoxifies poisonous compounds [50, 66]. Other ethnobotanical studies conducted within the country unveiled the most remedies to be prepared through decoction [17, 25, 67, 68]. This suggests that perhaps there is an unceasing exchange of information on the use of MHs among Tanzanian communities.

Most remedies were orally prescribed (96.6%), followed by topical (3.4%) (Table 2). The prevalence of oral administration can be described by a high frequency of internal illnesses in the area. Oral administration of herbal remedies is the most suitable pathway for ensuring a swift recovery and averting ailment attacks [69, 70]. The preponderance of oral administration for the diverse traditional remedies in the study area agrees with other ethnobotanical studies in Tanzania [25, 67].

### 3.10. Conditions of Remedy Preparations

In the present study, most herbal remedies (78.6%) in the study area are formulated from fresh plant materials, followed by dried form (19.2%), and 2.2% are prepared from fresh or dried plant materials. This observation specifies that fresh medicinal plant materials are considerably much easier and faster to formulate for remedy than other forms. A similar ethnobotanical study conducted in the Tabora Region [12] revealed that 85.2% of the herbal remedies were prepared from fresh plant materials, followed by dry form (14.8%). The locals' preference for fresh plant materials is primarily due to their efficacy in managing ailments since it is believed that the healing ingredients are preserved before utilization compared to the dried form. Usually, dried forms drop or lose their effectiveness due to the disappearance of some active compounds during the process [71].

## 4. Novelty

The recorded MH information was compared with other published works in online databases such as PubMed, Scopus, Web of Science, BioMed Central, and Google Scholar for novel uses. The data revealed notable differences in MH usage for different administrative modes. Out of 30 MHs recorded in the present study, five species were reported with new therapeutic usages for the first time. These MHs and their uses include the following: *Helianthus annuus* L. (Apocynaceae) (chest pain), *Catharanthus roseus* (L.) G. Don (Apocynaceae) (hypertension), *Helichrysum schimperi* (Sch. Bip. ex A. Rich.) Moeser (Asteraceae) (stomach ache and diarrhoea), *Maerua edulis* (Gilg & Gilg-Ben.) DeWolf (Capparaceae) (asthma), and *Satureja biflora* (Buch. Ham. ex D. Don) Briq. (Lamiaceae) (mental illness). It has been observed that these five species have been less explored pharmacologically to date. Thus, they should be subjected to phytochemical studies to authenticate their traditional uses.

## 5. Conclusion

The study area was rich in herbal medicine and corresponding indigenous knowledge diversity. Informants showed varying degrees of knowledge of medicinal plant use based on differences in gender, age, and education. More ethnobotanical knowledge was observed in females than males, elderly informants than younger ones, and illiterate than literate. Roots were vastly utilized to prepare various herbal remedies, but preferential use could harm the mother plants. Thus, using leaves as an alternative is highly encouraged for the conservation and sustainability of MHs. The higher IAR, UVs, and FLs of the recorded MHs can be used to empower future studies on pharmacology, phytochemistry, and conservation practices. Thus, consideration for the conservation of MHs and associated indigenous knowledge in the study area should be prioritized for their sustainability.

## Figures and Tables

**Table 1:** Demographic profile of informants and number of medicinal herbs.

**Variables**	**Categories**	**Number of informants**	**Percent (%)**	**Number of plants (± SD)**	**t**/**F -value**	**p** ** -value**
Gender	Male	42	42	7.4 ± 2.9^a^	−4.606	0.001
	Female	58	58	10.1 ± 3.0^b^		

Age groups (years)	< 20	12	12	5.6 ± 1.8^a^	22.984	0.001
	20–40	23	23	7.0 ± 1.3^a^		
	41–60	29	29	8.7 ± 2.4^bc^		
	> 60	36	36	11.0 ± 2.8^c^		

Education level	Illiterate	44	44	10.4 ± 3.0^a^	9.630	0.001
	Primary	30	30	9.2 ± 2.2^ab^		
	Secondary	17	17	7.5 ± 1.9^bc^		
	Tertiary	9	9	6.2 ± 2.2^c^		

*Note:* Different superscripts represent significantly different variable categories; *t*-value from the *t*-test and *F*-value from one-way ANOVA.

**Table 2:** List of medicinal herbs used by the Luguru tribe to treat various ailments.

**Family/scientific name (voucher no.)**	**Local name**	**So**	**PU**	**Ailment cured**	**MoP & RoA**	**UV**	**FUV**
Acanthaceae							0.700
*Justicia heterocarpa* L. (UL032)	Mwidu	W, C	R, L	Malaria	Crushing; oral	0.210	
Alliaceae							0.767
*Allium ascalonium* L. (UL041)	Kitunguu swaumu	C	R, L	Skin infection, fever	Decoction; oral	0.140	
*Allium sativum* L. (UL018)	Kitunguu maji	C	L	Malaria, fever, asthma, hypertension, cough	Concoction; oral	0.200	
Apocynaceae							0.600
*Catharanthus roseus* (L.) G. Don (UL077)	Myerusalemu	W, C	R, L	Hypertension, diabetes mellitus	Decoction; oral	0.180	
Asteraceae							2.233
*Bidens pilosa* L. (UL004)	Nyaweza	W, C	B	Wound, malaria, headache, kidney problems	Decoction; oral	0.430	
*Crassocephalum montuosum* v(S. Moore) Milne-Redh. (UL005)	Nyaluganza	W	B	Earache, headache, burn	Crushing; oral	0.230	
*Helianthus annuus* L. (UL003)	Ufuta	C	L	Chest pain, asthma	Decoction; oral	0.120	
*Helichrysum schimperi* (Sch. Bip. ex A. Rich.) Moeser (UL014)	Lweza	W	R	Stomach ache, diarrhoea	Decoction; oral	0.140	
*Sonchus pinnatifidus* L. (UL033)	Sungasunga	W	R, L	Stomach ache, headache	Decoction; oral	0.170	
*Vernonia amygdalina* Del. (UL007)	Msungu	W	R, L	Fever, diarrhoea, malaria, diabetes mellitus, headache, joint pain	Infusion; oral	0.570	
*Vernonia iodocalyx* O. Hoffm. (UL011)	Kitugutu	W	B, L	Stomach ache, diarrhoea, headache	Crushing; oral	0.230	
Capparaceae							0.333
*Maerua edulis* (Gilg & Gilg-Ben.) DeWolf (UL075)	Kilialia	W, C	R, L, F	Aphrodisiac, asthma, venereal diseases	Crushing; oral	0.070	
*Cucurbita moschata* Duchesne. (UL043)	Maboga	C	R	Menstrual disorders	Infusion; oral	0.060	
Euphorbiaceae							0.933
*Acalypha fruticosa* Forssk. (UL029)	Kifulwe	W	L	Diarrhoea, rheumatism, dyspepsia, skin infections	Decoction; oral	0.280	
Fabaceae							1.167
*Abrus precatorius* L. (UL079)	Lufambo	W	R	Diarrhoea, eye inflammation, women's infertility, aphrodisiac, wounds, sores, cough	Decoction; oral	0.320	
*Cassia mimosoides* L. (UL057)	Lusangalala	W	R, B	Asthma, mental illness, cough	Decoction; oral	0.130	
*Mucuna pruriens* (L.) DC (UL067)	Bumu	W	R, Se	Aphrodisiac, nervine tonic, anti-inflammatory	Infusion; oral	0.240	
*Vigna unguiculata* (L.) Walp. (UL063)	Mkunde	C	R, L	Cough, chest pain, abscess	Infusion; oral	0.150	
Lamiaceae							1.533
*Ocimum americanum* L. (UL047)	Mvumbasi	W, C	R	Epilepsy, ulcers, stomach ache, pneumonia	Decoction; oral	0.150	
*Ocimum suave* Willd. (UL0002)	Mnung'ha	W, C	B	Malaria, stomach ache	Decoction; oral	0.370	
*Satureja biflora* (Buch. Ham. ex D. Don) Briq. (UL012)	Luparalwa mlungu	W	L	Mental illness	Infusion; oral	0.180	
Melastomataceae							0.633
*Dissotis rotundifolia* (Sm.) Triana. (UL040)	Kinzasu	W	R, L	Asthma, gonorrhoea, wound, boil, abscess, diarrhoea, malaria	Crushing; oral	0.190	
Smilacaceae							0.300
*Smilax anceps* Wild. (UL046)	Mkwangasale	W	L	Tuberculosis	Decoction; oral	0.090	
Solanaceae							1.100
*Capsicum frutescens* L. (UL008)	Pilipililukwale	C	R, B	Skin infections, wound	Crushing; topical	0.110	
*Lycopersicum esculentum* Mill. (UL106)	Mnyanya	C	R, L	Stomach ache	Concoction; oral	0.170	
*Physalis peruviana* L. (UL031)	Nkuluwanti	W	R, L	Malaria, typhoid,	Decoction; oral	0.100	
*Nicotiana tabacum* L. (UL107)	Tumbaku	C	F	Hernia	Decoction; oral	0.070	
*Solanum incanum* L. (UL108)	Mtula	W, C	L	Cough, vomit.	Concoction; oral	0.190	
Zingiberaceae							0.700
*Zingiber officinale* Roscoe (UL006)	Tangawizi	C	R	Cough, constipation, asthma	Decoction; oral	0.210	
Zygophylaceae				Toothache	Chewing; oral		0.400
*Tribulus terrestris* L. (UL038)	Mbigiri nene	W	L	Gonorrhoea, chest pain, earache	Decoction; oral	0.060	

Abbreviations: B, bark; C, cultivation; F, fruit; FUV, family use value; L, leaf; MoP, mode of preparation; PU, part used; R, root; RoA, route of administration; Se, seed; So, source; UR, use reports; UV, use value; W, wild.

**Table 3:** Fidelity levels of medicinal herbs commonly reported against human ailment category.

**Medicinal herb**	**Ailment category**	**Ip**	**Iu**	**FL**
*Justicia heterocarpa*	Malaria	21	21	100
*Catharanthus roseus*	Hypertension	18	18	100
*Cucurbita moschata*	Menstrual disorders	6	6	100
*Acalypha fruticosa* Forssk.	Rheumatism and skin infections	28	28	100
*Dissotis rotundifolia*	Gonorrhoea and diarrhoea	19	19	100
*Smilax anceps*	Tuberculosis	9	9	100
*Vernonia amygdalina*	Diabetes mellitus, headache, and joint pain	56	57	98
*Abrus precatorius*	Diarrhoea, aphrodisiac, and cough	29	32	91
*Allium sativum*	Fever and cough	17	20	85
*Ocimum suave*	Stomach ache	30	37	81

**Table 4:** Informant agreement ratio (IAR) and ailment categories.

**Ailment category**	**List of plant species and number of use reports**	**Nt**	**Nur**	**IAR**
Malaria and fever	*Justicia heterocarpa* (21), *Allium ascalonium* (9), *Allium sativum* (16), *Bidens pilosa* (36), *Vernonia amygdalina* (46), *Ocimum suave* (28), *Dissotis rotundifolia* (10), *Physalis peruviana* (10)	8	176	0.96
Gastrointestinal disorders	*Helichrysum schimperi* (14), *Sonchus pinnatifidus* (14), *Vernonia amygdalina* (40), *Vernonia iodocalyx* (17), *Acalypha fruticosa* (22), *Abrus precatorius* (11), *Ocimum americanum* (12), *Ocimum suave* (17), *Lycopersicum esculentum* (3), *Physalis peruviana* (1), *Solanum incanum* (5), *Zingiber officinale* (8)	12	164	0.93
Respiratory disorders	*Allium sativum* (8), *Helianthus annuus* (7), *Maerua edulis* (2), *Cassia mimosoides* (9), *Vigna unguiculata* (5), *Ocimum americanum* (9), *Dissotis rotundifolia* (3), *Smilax anceps* (9), *Solanum incanum* (1), *Zingiber officinale* (20)	10	73	0.88
Dermatological disorders	*Allium ascalonium* (6), *Bidens pilosa* (23), *Crassocephalum montuosum* (5), *Acalypha fruticosa* (7), *Abrus precatorius* (4), *Dissotis rotundifolia* (3), *Capsicum frutescens* (11)	7	59	0.90
Reproductive disorders	*Maerua edulis* (4), *Abrus precatorius* (13), *Cassia mimosoides* (2), *Cucurbita moschata* (6), *Mucuna pruriens* (17), *Nicotiana tabacum* (7)	6	49	0.90
Metabolic disorders	*Allium sativum* (5), *Catharanthus roseus* (18), *Vernonia amygdalina* (13)	3	36	0.94
Neurological disorders	*Ocimum americanum* (3), *Cassia mimosoides* (3), *Satureja biflora* (18), *Mucuna pruriens* (2)	4	32	0.90
Pains/sprain	*Crassocephalum montuosum* (3), *Vernonia amygdalina* (7), *Zingiber officinale* (4), *Tribulus terrestris* (4)	4	18	0.82
Sexually transmitted infections	*Dissotis rotundifolia* (7), *Tribulus terrestris* (3)	2	10	0.89

## Data Availability

The data that support the findings of this study are available from the corresponding author upon reasonable request.
